# The Impact of Different Parental Figures of Adolescents Living With HIV: An Evaluation of Family Structures, Perceived HIV Related Stigma, and Opportunities for Social Support

**DOI:** 10.3389/fpubh.2022.647960

**Published:** 2022-03-24

**Authors:** Gloria Wowolo, Wangnan Cao, Dennis Bosomtwe, Anthony Enimil, Nicholas Tarantino, David H. Barker, Omar Galárraga

**Affiliations:** ^1^Brown University School of Public Health, Providence, RI, United States; ^2^Department of Health Services, Policy and Practice, Brown University School of Public Health, Providence, RI, United States; ^3^Department of Child Health, Komfo Anokye Teaching Hospital, Kumasi, Ghana; ^4^Department of Child Health, Kwame Nkrumah University of Science and Technology, Kumasi, Ghana; ^5^Department of Psychiatry, Rhode Island Hospital, Providence, RI, United States; ^6^Department of Psychiatry and Human Behavior, Brown University Alpert Medical School, Providence, RI, United States

**Keywords:** stigma, HIV, adolescents, support, single mothers, Ghana, family

## Abstract

Although antiretroviral therapy (ART) has changed the expected health outcomes for HIV, there are still issues related to stigma, how people living with HIV are perceived, and the availability of social support. The purpose of this study was to explore the associations between family structure and psychosocial wellbeing reflected by perceived HIV stigma and social support among adolescents living with HIV in Kumasi, Ghana. This article used baseline data from two mixed methods studies that evaluated the safety and preliminary efficacy of group-based support programs for ART adherence improvement among adolescents living in Kumasi, Ghana (*N* = 70, aged 12–18 years). A multivariate linear regression analysis was employed to examine the associations between family structure and the outcomes of stigma and social support. The main variables for family structure were single mothers and female caregivers. We found that single motherhood was a significant determinant of stigma. When compared to other categories of caregiver types, adolescents being raised by their single mothers was associated with a 0.259 decrease in the mean internal HIV stigma score (*p* = 0.029). Also, for female adolescents, being raised by a female guardian (e.g., mother, aunt, grandmother, and sister) was associated with a 20.92 point increase in the overall support index (*p* = 0.005). This study shows that the type of parent or guardian, and their gender, influences the perceived stigma and available social support among adolescents living with HIV in Ghana. Vulnerable subgroups of adolescents living with HIV, particularly those raised up by male caregivers, should be provided with additional support.

## Introduction

An estimated 1.7 million adolescents (10–19 years old) were living with HIV globally in 2019 (1), and 84% of them were living in sub-Saharan Africa (2). Given the advancements in medicine, adolescents with perinatally acquired HIV have the ability to manage their physical health outcomes, but social outcomes may be harder to navigate. Generally, while work has been done to develop support for this population, there are still gaps in knowledge around best practices related to adolescents living with HIV (3, 4). Adolescence is a formative developmental period and HIV-related stigma has prominent influence on how adolescents choose to manage their health and navigate their social relationships (5). At the same time, familial relationships also affect adolescents' view of their disease, especially considering how their HIV positive parents chose to navigate stigma and disclosure (6). Given the various factors that can influence the lives of adolescents living with HIV, it is important to investigate potential barriers to their wellbeing, as these obstacles may also impact their continuation of care.

Social perception of HIV continues to be a prominent issue for health and social outcomes for those living with HIV. For adolescents living with HIV (ALHIV), there is the double burden of enacted stigma and internalized stigma especially if their environment is hostile or discriminatory against people with a positive HIV serostatus. Recent studies show that adolescents living with HIV who have negative societal interactions have a high risk of depressive disorder and suicidality (7).

In Ghana, the median prevalence of HIV infection is estimated to be 1.7% among adults (aged 15–49 years) and 0.7% among adolescents (aged 15–19 years). Of the 18,928 new HIV infections that occurred in the country in 2020, 1,985 (10%) and 5,211 (27.5%) were in adolescents and young adults aged 10–19 and 15–24 years, respectively (3, 8, 9). HIV care in Ghana is hospital-based, with urban facilities staffed by medical officers and clinical specialists and rural and community-based centers relying on physician assistants and nurse practitioners. HIV treatment centers predominately focus on addressing pediatric and adult care needs; very few clinics in Ghana are equipped to offer services that address the specific clinical and psychosocial needs of adolescents living with HIV. This is largely because allocating additional financial and human resources (e.g., social support groups and trained adolescent psychiatrics) to operate an adolescent clinic is often outside the capacity of district hospitals that are already caring for children and adults living with HIV within a limited budget. Because the time period for which patients require adolescent-specific services before aging into adulthood is relatively short, expanding such services for ALHIV would need to prove to be cost-effective for facilities with limited resources; and to the best of our knowledge such evidence is not yet available. The limited availability of adolescent-specific care for ALHIV in Ghana is compounded by persistent HIV-related stigma and other barriers to treatment adherence, which contribute to worse HIV-related outcomes in this population: 67% of ALHIV in Ghana are not virally suppressed compared to 7–53% of ALHIV in neighboring sub-Saharan African countries (10).

Given the emphasis on childrearing and familial systems in Ghana, understanding how those ties possibly relate to HIV health outcomes could be an innovative way to view treatment (10, 11). HIV stigma in the communities and even amongst close family members is high. Many adolescents in Ghana therefore learn about and manage their HIV diagnosis through one primary caregiver, thus this study provides an opportunity to understand how that relationship can impact one of the most vulnerable groups in Ghana (12). When considering how to address the issues adolescents face, it is important to fully understand the role and impact of caregivers. Emphasizing the connection between available social support and an ALHIV's mental or emotional health could be instrumental in creating relevant support opportunities outside of clinical care (13, 14). Investigating the possible connections to family structure as it relates to an adolescent living with HIV's experiences with stigma and social support might allow for a better understanding of how to fully engage this population in their HIV care.

It has been well documented that low stigma and high social support were linked to better clinical outcomes among people living with HIV (4, 5), such as good treatment adherence and high viral suppression (15, 16). One determinant of psychosocial wellbeing reflected by stigma and social support was family background/structure (6, 7). However, most of these studies were conducted in high-income countries that have different culture and economic backgrounds compared to Ghana. Local studies that are focusing on Ghana ALHIV are lacking.

The purpose of this study was to explore the associations between family structure (with regard to the type and gender of the caregiver) and perceived HIV stigma and social support among adolescents living with HIV in Kumasi, Ghana. We hypothesized that adolescents living with HIV who were taken care of by mothers (vs. others) or by female caregivers (vs. male caregivers) would have better psychosocial wellbeing reflected by low stigma and high social support.

## Methods

### Setting and Population

The data collection for this study took place in Kumasi, Ghana; we used information from two feasibility studies of providing peer support groups during regular clinic visits. Sample size for each study was determined by the practical constraints of running 5 in-clinic peer groups of 6 to 8 participants. Each study was conducted over the course of 9 months in 2015 and 2017. The adolescent HIV clinic at the Komfo Anokye Teaching Hospital (KATH) was the main research site for both studies. Data was collected by a Ghanaian research team led by Dr. Anthony Enimil through a partnership with Dr. David Barker and Dr. Omar Galárraga. Both studies assessed the impact of in-clinic support groups on engagement in care. The study protocols were approved by institutional review boards of KATH, Kumasi, Ghana (CHRPE/AP/314/14, CHRPE/373/17), Rhode Island Hospital, Providence, RI (FWA00001230), and Brown University (1707001848, FWA 00004460). Each study recruited 35 adolescent patients from the clinic with no overlap in recruitment). Criteria for study inclusion were: (a) 12–19 years of age, (b) perinatally-acquired HIV infection (confirmed by DNA/RNA polymerase chain reaction PCR), (c) on ART and had a detectable viral load at baseline, (d) willingness to consent to participation. Participants with advanced illness that impaired cognitive ability to provide informed consent were excluded. Results from both studies have been published (17, 18). Neither set of results examined the impact of gendered family structures (independent variable) on adolescents living with HIV and their perceived HIV related stigma and available social support (dependent variables), which is the unique contribution of this article. Recruitment was during routine clinic visits at KATH adolescent clinic, which provided care to 180 ALHIV at the time of the study. All clinical visits were held in the morning, and the adolescent patients were seen by doctors on a first-come-first-served basis. For participants younger than 18 written parental consent and participant assent were obtained. Participants 18 years of age provided written consent.

### Research Design, Data Collection, and Management

The quantitative assessments for both feasibility studies were conducted at baseline and during 3-, 6-, and 9- month follow up visits. For this cross-sectional analysis, we analyzed data that were collected at baseline from 70 adolescent patients receiving HIV-related service at a local clinic. In the present analysis, the outcomes of interest were (1) perceived HIV-related stigma and (2) perceived social support. HIV-related stigma was measured at baseline and each follow-up visit using the *Perceived HIV-Related Stigma Scale*, a 12-item scale that captures both internal and external HIV-related stigma (19). An example scale item was “Having HIV makes me feel I am a bad person.” Each item was rated on the 4-point scale from strongly disagree (1) to strongly agree (4). Scores were computed by separately averaging participant responses for the internal and external stigma scales; for both scales, higher scores indicated more stigma. For this study, total scores for the overall scale were not used. Cronbach α for the internal stigma scale and externalized stigma scale were 0.76 and 0.83, respectively. Perceived social support was measured using the *Medical Outcomes Study (MOS) Social Support Survey* (20). The survey assessed individuals' perceptions about available emotional/informational support (8 items), tangible support (4 items), affectionate support (4 items), and positive social interaction (4 items). Each item was rated on a 5-point scale from none of the time (1) to all of the time (5). The overall support index was calculated and transformed to a 0–100 scale. The Medical Outcomes Study was specifically developed for patients with chronic conditions to evaluate functional social support. Cronbach α was 0.87.

The independent variables were coded as categorical in order to differentiate between caregiver type. Caregiver type was broken down into two different groups, single mothers vs. other caregivers or female caregivers vs. other caregivers. For the single mother group, only the response of mother was included in this classification, the other caregivers included responses of both parents, father, aunt, uncle, grandmother, stepmother, sister, and brother. The category of female caregiver included mother, aunt, grandmother, sister, and stepmother while the other caregivers included their male counterparts. For the covariates of sex and age, sex was categorized as a binary variable (male/female), while age was grouped as a categorical variable of 12–14 years old, 15–16 years old, and 17–18 years old. We chose to categorize age by early, mid and late adolescence to better reflect developmental changes, which can follow non-linear patterns.

### Statistical Analysis

Since both studies used the same measures and followed similar procedures, data harmonization was not required prior to pooling the data across study. There was no missing data because only baseline information was included in this analysis. Summary statistics were calculated with frequency (%) for sex and age. Multivariate linear regression (MLR) models were used to examine associations between family structure and the primary outcomes, stigma and social support. First, we estimated the mean score of stigma and social support between participants who were taken care of by a single mother and those taken care of by others while controlling for sex and age. Second, we compared the mean score of stigma and social support between participants who were taken care of by female caregivers and those taken care of by male caregivers while controlling for sex and age. MLR models were computed separately for stigma and social support to describe the linear relationship between the multiple predictor variables (family structure attributes) for the prediction of a single dependent variable (*Osi* (stigma) or *Ossi* (social support) coefficients), as is in Equation (1):


(1) $$\begin{array}{r} O_{\mathrm{si}}={\text{β}}_{0}+{\text{β}}_{1i}X_{1i}+{\text{β}}_{2i}X_{2i}+\ldots +{\text{β}}_{\mathrm{ni}}X_{\mathrm{ni}}+{\text{ε}}_{i} \end{array}$$

where *Os*_*i*_ is the stigma or social support coefficient, β_0_ is the corresponding intercept term, β_1i_, β_2i_, and β_*ni*_ are the prediction coefficients, *X*_1i_, *X*_2i_, and *X*_*ni*_ are the independent predictor variable values for the *i*^th^ *Os*_*i*_ or *Oss*_*i*_ coefficients for the *n*^th^ family structure attributes, and ε*i* is the random error term. Statistical significance was set at an α of 0.05.

All analyses were conducted using Stata SE16.1 (21).

## Results

Among the 70 total participants, adolescents ages 12–14 years old consisted of 51.4% of the sample, 15–16 years old at 32.8%, and 17–18 years old at 15.7% (Table 1). Although clinic patients 12–19 years of age were eligible to enroll in the feasibility studies, none of the 70 participants who consented to participate were older than 18 years of age. More than half of the participants (55.7%) were male (Table 1). For the caregiver types, single mothers made up 35.7% of the group while female caregivers represented 51.3% of the group when compared to their pairings (Table 1). For all participants regardless of caregiver type, the average internalized stigma score was 1.97 (SD: 0.483), while the average externalized stigma score was 2.48 (SD: 0.526), and the mean MOS total support score was 66.4 (SD: 15.57) (Table 1).

**Table 1:** Characteristics of study sample.

**Mean (SD) or *n* (%)**	***n*** **= 70**
**Sex**	
Male	39 (55.71%)
Female	31 (44.29%)
**Age**	
12–14 years old	36 (51.43%)
15–16 years old	23 (32.86%)
17–18 years old	11 (15.71%)
**Caregiver type 1**	
Single mother	25 (35.71%)
Other (e.g., father, aunt, etc.)	45 (64.29%)
**Caregiver type 2**	
Female caregiver	36 (51.43%)
Other (e.g., male caregiver)	34 (48.57%)
**Perceived stigma**	
Internalized stigma (1–5)	1.97 (0.483)
Externalized stigma (1–5)	2.48 (0.526)
**Social support**	
MOS total support (0–100)	66.37 (15.57)

*MOS, Medical Outcomes Study; SD, standard deviation*.

Adolescents who are being raised by their single mothers had an average internal stigma score of 1.81 while adolescents being raised by others (e.g., grandmother, uncle, father, and aunt) scored an average of 2.06 (see Table 2 for disaggregated data). This corresponds to a 0.259 lower mean internal stigma score (*p* = 0.029) among adolescents being raised by single mothers compared to others caregivers, and indicates moderate associations between caregiver type and perceived HIV-related stigma.

**Table 2:** Mean scores for outcomes by caregiver type—single mother.

	**Internalized stigma**	**Externalized stigma**	**MOS total social support**
**Single mother**			
Male	1.77 (0.45)	2.29 (0.49)	50.96 (19.58)
Female	1.86 (0.44)	2.52 (0.51)	74.54 (12.37)
12–14 years old	1.67 (0.43)	2.32 (0.56)	61.84 (18.68)
15–16 years old	2.00 (0.35)	2.40 (0.53)	68.94 (13.94)
17–18 years old	1.87 (0.55)	2.57 (0.35)	73.16 (22.88)
**Other caregiver (e.g., both parents, father, aunt, uncle, grandmother, etc.)**			
Male	1.97 (0.48)	2.55 (0.49)	66.58 (15.42)
Female	2.17 (0.49)	2.53 (0.61)	66.12 (12.98)
12–14 years old	1.96 (0.36)	2.38 (0.42)	65.63 (14.03)
15–16 years old	2.10 (0.45)	2.52 (0.56)	66.05 (15.54)
17–18 years old	2.33 (0.91)	3.19 (0.46)	70.18 (13.41)

Age also appears to be moderately associated with external HIV-related stigma; it was found that older adolescents (17–18) scored higher when it comes to average external stigma when compared to their younger counterparts. Not accounting for caregiver type, participants ages 17–18 years old, had an average external stigma score of 2.91 while adolescents ages 12–14 years old had an average score of 2.36. For adolescents ages 17–18 years old who are being raised by their single mothers, there was a 0.569 increase in the mean external HIV stigma score (*p* = 0.001). Similar associations are seen for adolescents of the same age who are being raised by female caregivers, there was a 0.579 (*p* = 0.001) increase related to their external stigma scores (Table 3).

**Table 3:** Mean scores for outcomes by caregiver type—female caregiver.

	**Internalized stigma**	**Externalized stigma**	**MOS total social support**
**Female caregiver**			
Male	1.83 (0.54)	2.39 (0.57)	59.57 (17.87)
Female	1.96 (0.45)	2.55 (0.48)	73.79 (11.32)
12–14 years old	1.67 (0.42)	2.31 (0.56)	62.50 (16.99)
15–16 years old	2.08 (0.37)	2.43 (0.51)	65.81 (15.97)
17–18 years old	2.13 (0.65)	2.77 (0.41)	70.07 (19.17)
**Other caregiver (e.g., father, uncle, brother)**			
Male	1.98 (0.36)	2.55 (0.39)	70.20 (14.36)
Female	2.14 (0.52)	2.50 (0.64)	65.25 (13.71)
12–14 years old	2.06 (0.27)	2.41 (0.35)	66.23 (14.24)
15–16 years old	2.05 (0.45)	2.51 (0.58)	68.02 (14.34)
17–18 years old	2.11 (1.21)	3.28 (0.67)	75.44 (13.82)

**Figure 1:**
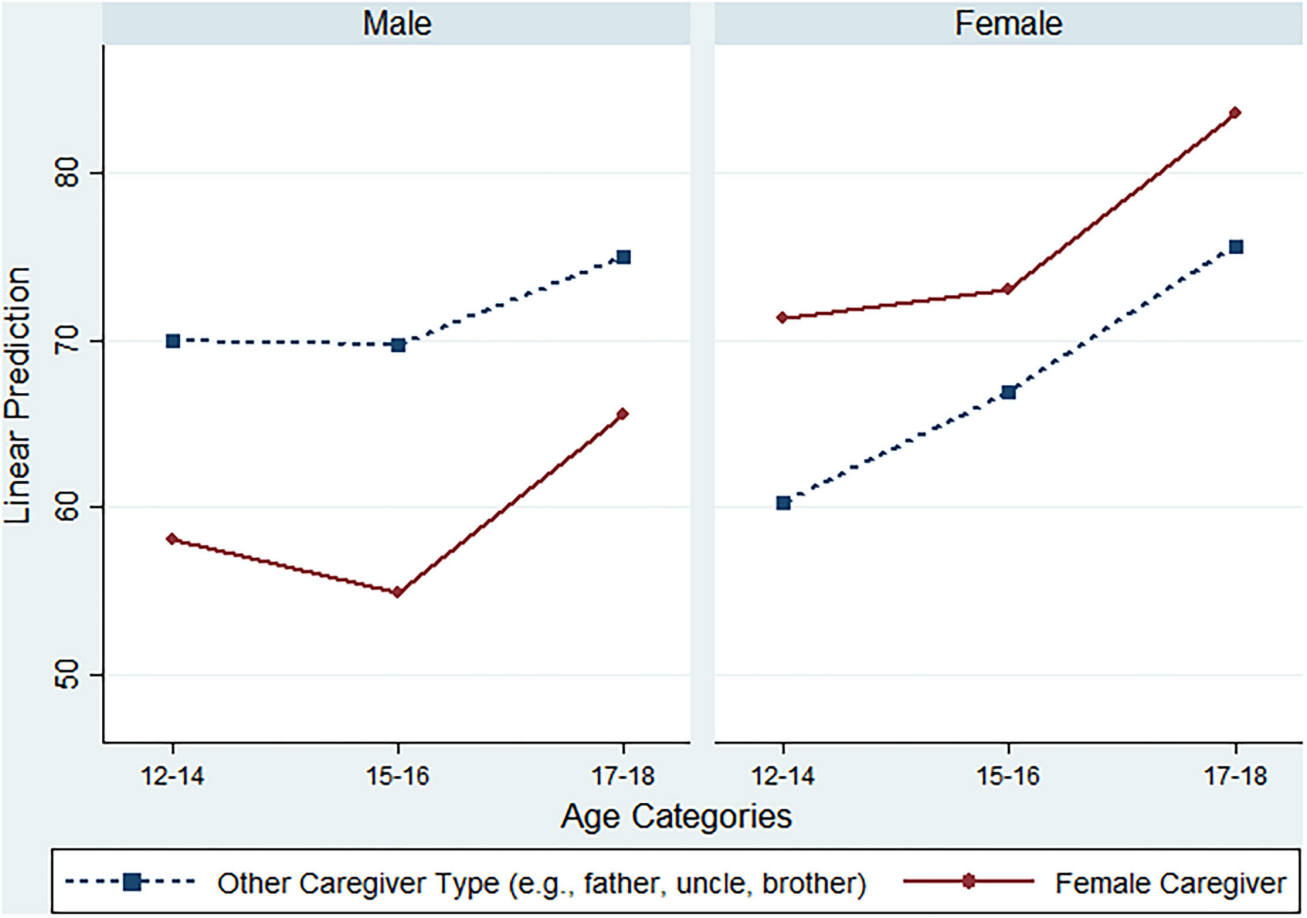
MOS total social support margins by caregiver type, sex, and age.

The interaction of gender and caregiver type produced significant results. For female adolescents, being raised by a female guardian (e.g., mother, aunt, grandmother, and sister) was strongly associated with a 20.92 point increase in the overall support index (*p* = 0.005). There were distinct differences related to the gender of a caregiver and the gender of an adolescent. Both male and female adolescents report higher total social support scores when being raised by a caregiver that matches their gender (Figure 1). All of this information shows that family and parental support is needed in order to address feelings of stigma and opportunities for support.

## Discussion

Although previous research has shown the significant need for improved support for adolescents living with HIV, this study identifies the overall importance of an adolescent's guardian type as it relates to their feelings of stigma and social support (22, 23). As an exploratory analysis, this study allows for a greater understanding of the possible impact of different guardians and highlights the need for more support for older adolescents. Given the pervasiveness of HIV related stigma, ALHIV are at risk of experiencing external stigma in almost all areas of their lives, while also struggling to address their feelings of internal stigma (24). If more is not done to support this group, even with the medical advancements related to HIV treatment, there could be another generation lost to HIV due to the struggles with mental health many adolescents face.

Our findings indicate that being raised by a mother can reduce an adolescent's internalized stigma and increase their feelings of social support. Being raised by a caregiver of their own gender also seems to improve adolescents feelings of support. One possible explanation for these findings may be that having a caregiver of the same gender can increase feelings of mutual trust and shared understanding, which can help reduce common HIV-related burdens facing adolescents, such as the burden of dealing with HIV-related stigma and routinely taking HIV medications (25, 26). Additionally, it appears that adolescents in the 17–18 age range report higher external stigma scores when compared to their younger counterparts, which was echoed with previous studies reporting that Ghana adolescents aged 17–18 years had the lowest low-esteem (27). High stigma and low esteem demonstrated their dual vulnerability, which has implications for the need to strengthen Ghana's mental health program implementation particularly for adolescents living with HIV.

The present study found a high-level stigma among adolescents living with HIV in Ghana, which was consistent with prior studies (12, 28–30). Experiences of stigma, as well as the fear of being stigmatized had implications for clinic attendance and treatment and therefore should be recognized and addressed to better support adolescents living with HIV. These stigma concerns faced by adolescents living with HIV are a result of the inherent beliefs of the Ghanaian society about HIV, such as a person with HIV infection is perceived to be immoral and anyone living with HIV is dangerous and must be isolated from society (31). The high-level of stigma also suggested the need for psychological support for both adolescents living with HIV and their family members. In addition to healthcare professionals, social workers would be able to play a crucial role in improving the psychosocial welling of these adolescents patients (12, 26).

This study has some limitations. First, the sample size was relatively small, which yielded limited power in detecting statistically significant differences in outcomes of stigma and support varied by caregiver type and gender. Second, the age cutoffs were a bit arbitrary. Third, there might be other confounders we did not measure and adjust in terms of the association between family structure and stigma/social support. Also, the original studies were not developed to investigate the impact of parental type on feelings of stigma and social support, but this study was able to derive information based on the available study questions.

To our knowledge, this is one of the first studies investigating how family structure would affect stigma and social support among adolescent living with HIV in the Ghana setting. Stigma and social support were measured with validated scales documenting good internal reliability in this less-investigated population in Ghana. The study findings contribute to the literature on adolescents living with HIV and shed new light on the influence of family structures. Specifically looking at single mothers and female caregivers allows for more knowledge to be built around how this population fares when raising adolescents living with HIV. Studies have shown that family-based programs for ALHIV and their caregivers are useful in supporting mental health challenges, increasing adherence to medicine, and reducing feelings of stigma (26, 32). Supporting caregivers of ALHIV in Ghana as they support and take care of their children, would be a way to address some of the most prominent needs of this population and improve some of their social and clinical health outcomes (32, 33). Although the sample size for this study was small, the findings are a start into developing more comprehensive treatment programs for ALHIV.

By dedicating resources to increase support to caregivers of adolescents living with HIV, the field of public health would be investing in the lives of patients and expanding what is considered to be HIV treatment. Although clinical outcomes are important, failing to treat the whole person or not creating opportunities to support mental health needs could be just as detrimental as not being able to access HIV medication. This is an opportunity to go beyond the usual scope of treatment and develop programs that fully support adolescents and their caregivers.

## Conclusion

The present study focused on adolescents living with HIV, who are highly stigmatized in Ghana. We found that adolescents who were taken care of by mothers (vs. others) or by female caregivers (vs. male caregivers) had better psychosocial wellbeing reflected by low stigma and high social support. Vulnerable subgroups of adolescents living with HIV, particularly those raised up by male caregivers, should be provided with additional support. We conclude that family structure plays an important role in the adolescents' psychosocial wellbeing in a resource-limited setting.

## Data Availability Statement

The data analyzed in this study is subject to the following licenses/restrictions: requests to access these datasets should be directed to GW, gloria_wowolo@alumni.brown.edu.

## Ethics Statement

The studies involving human participants were reviewed and approved by Institutional Review Boards of KATH, Kumasi, Ghana (CHRPE/AP/314/14, CHRPE/373/17), Rhode Island Hospital, Providence, RI (FWA00001230), and Brown University (1707001848, FWA 00004460). Written informed consent to participate in this study was provided by the participants' legal guardian/next of kin.

## Author Contributions

GW conceived and planned the study, analyzed the data, and wrote with input from OG and DHB. DB, WC, and NT supported in the data cleaning and organization process. AE, OG, and DHB planned the original studies that this data is based on. All authors contributed to the article and approved the submitted version.

## Funding

This research was funded by the Brown University Global Public Health Program.

## Conflict of Interest

The authors declare that the research was conducted in the absence of any commercial or financial relationships that could be construed as a potential conflict of interest.

## Publisher's Note

All claims expressed in this article are solely those of the authors and do not necessarily represent those of their affiliated organizations, or those of the publisher, the editors and the reviewers. Any product that may be evaluated in this article, or claim that may be made by its manufacturer, is not guaranteed or endorsed by the publisher.
